# The impact of parental co-parenting on relational aggression in adolescents: the mediating role of peer relationships and self-control

**DOI:** 10.3389/fpsyg.2025.1551288

**Published:** 2025-04-22

**Authors:** Yongzhi Jiang, Huizhe Wang, Lifang Tong

**Affiliations:** ^1^School of Educational Science, Inner Mongolia Minzu University, Tongliao, China; ^2^Inner Mongolia Autonomous Region Student Bullying Prevention and Control Research Center, Tongliao, China

**Keywords:** parental co-parenting, peer relationships, self-control, relational aggression, adolescents

## Abstract

**Purpose:**

To explore the impact of parental co-parenting on adolescents’ relational aggression, as well as the mediating roles of peer relationships and self-control.

**Method:**

A self-reported questionnaire survey was conducted on 550 students from three junior high schools in Inner Mongolia using the Parental Coordinated Parenting Scale, Relational Aggression Scale, Self-Control Scale, and Peer Relationship Scale.

**Result:**

Positive parental co-parenting is significantly negatively correlated with relational aggression, while negative co-parenting is significantly positively correlated with relational aggression. Positive co-parenting is significantly positively correlated with peer relationships and self-control, while negative parental co-parenting is significantly negatively correlated with peer relationships and self-control. Relational aggression is significantly negatively correlated with peer relationships and self-control, and peer relationships are significantly positively correlated with self-control. The direct effect of negative parental co-parenting on relational aggression is significant, and self-control, peer relationships, and the mediating effects of peer relationships and self-control in the relationship between parental co-parenting and relational aggression are all significant.

**Conclusion:**

Parental co-parenting not only directly affects adolescents’ relational aggression but can also indirectly influence relational aggression through the mediating effects of peer relationships and self-control.

## Introduction

Campus violence and bullying have always been issues of concern for schools, families, and society. Relational aggression is a common form of campus attack that is also covert and difficult to detect, but it can have a significant impact on the physical and mental health of adolescents. Co-parenting is an essential part of family education and has a significant influence on the formation and development of adolescents’ personality, cognition, and character. At the same time, self-control is part of the cognitive development of students, and co-parenting plays an important role in the development of children’s self-control. Therefore, parenting styles can influence students’ self-control, which in turn affects relational aggression behavior. Additionally, peers are an indispensable part of adolescents’ learning and life, and parenting styles can also influence students’ peer relationships, thereby affecting relational aggression behavior. This study mainly explores the impact of co-parenting on adolescents’ relational aggression, as well as the mediating roles of peer relationships and self-control.

Co-parenting refers to the collaborative activities of adults who share the responsibility of raising and nurturing children during the child-rearing process (McHale, 2007). In most families, co-parenting is generally composed of parents; the higher the level of cooperative behavior between parents, the more positive children perceive their parents’ relationship, thereby increasing their sense of security and reducing anxiety (Metz et al., 2017). Minuchin (1974) believes that parents’ co-parenting to regulate family interactions contributes to the development of each family member, and the overall intimacy level and relative intimacy patterns of family relationships play a very important role in the overall functioning of the family system and the physical and mental health of its members. Researchers have different classifications of co-parenting, such as cooperative, conflictual, and triangular relationships (Margolin et al., 2001), and other classifications based on different dimensions include unity, consistency, conflict, and belittling (McHale, 1997). Family education is the first education an individual receives; therefore, parents’ parenting styles play a primary role and have a significant impact on children’s growth and development. Existing research shows that parents’ relevant behaviors and parenting styles are related to individuals’ relational aggression behaviors, and good co-parenting practices between parents help individuals develop at different stages (McHale, 2011). Smith found that parental phubbing (excessive smartphone use in children’s presence) significantly positively predicted adolescents’ engagement in cyber-relational aggression, highlighting the role of digital neglect in modern parenting (Smith et al., 2022). Parents adopting a supportive co-parenting approach during the child-rearing process can significantly reduce relational aggression behaviors in middle school students (Xu, 2019). Additionally, Inner Mongolia’s unique socio-cultural landscape, blending Han Chinese and ethnic Mongolian traditions, may influence co-parenting practices. “In Inner Mongolia, where cultural norms prioritize familial cohesion, covert relational aggression may serve as a socially sanctioned alternative to overt confrontation, necessitating region-specific investigations.” For instance, “Within Inner Mongolia’s cultural matrix where Han Chinese agrarian traditions interface with Mongolian pastoralist norms (Hofstede et al., 2021), co-parenting practices exhibit unique conflict regulation patterns.

### Parental co-parenting and relational aggression

Relational aggression refers to behaviors in which individuals intentionally harm others as a primary goal, manipulating or damaging interpersonal relationships through means such as defamation, isolation, and gossip (Crick and Grotpeter, 1995). It has a certain degree of harm, but is not as direct and obvious as physical aggression, exhibiting a covert nature (Skowronski et al., 2005). Behaviors of relational aggression include withdrawing friendship, spreading rumors, and excluding someone from a group (Liu et al., 2022). Relational aggression is increasingly recognized as a covert form of harm with long-term psychological consequences, particularly in collectivist cultures where indirect aggression may be socially normalized (Li and Wang, 2021). According to social learning theory, the connection between parenting and relational aggression can be understood through imitation and vicarious reinforcement (Bandura et al., 1997). Children may imitate their parents’ use of relational aggression and spread rumors about their friends in social contexts, or they may learn some relational aggression behaviors from the negative co-parenting dynamics between their parents and implement these behaviors in their social interactions. Existing research shows that relational aggression occurs among same-sex peer relationships (Crick et al., 2006). Social exclusion can not only directly predict relational aggression but can also provoke relational aggression by threatening an individual’s self-esteem (Yujie et al., 2019). If relational aggression is not well managed, it may increase with age and become more covert (Wang and Tan, 2016). Individual factors, including physiological, emotional, cognitive, and temperamental aspects, can influence children’s aggressive behaviors (Cha, 2006). Family factors are also a major focus of many studies, the predictive role of parental psychological control on adolescent aggressive behavior (Jiang, 2016). Negative parenting styles contribute to aggressive behaviors in children and adolescents (Mukhtar and Mahmood, 2018). Family factors, such as co-parenting dynamics, are critical in shaping adolescents’ social behaviors. Recent studies highlight that negative co-parenting (e.g., conflict and belittling) amplifies Relational aggression by modeling manipulative interpersonal strategies (Liu et al., 2022). Therefore, good family parenting practices can help reduce aggressive behaviors in adolescents. Based on this, the study proposes hypothesis H1: Parental co-parenting can predict adolescents’ relational aggression behaviors.

### The mediating role of peer relationships

Peer relationships refer to the interpersonal relationships established or developed by individuals of similar age or comparable psychological development during their interactions. Compared to parent–child relationships and teacher-student relationships, peer relationships are equal and parallel (Zhou et al., 2015). After starting school, children’s attachment gradually shifts from parents to peers, while the parenting styles of children’s parents can influence their peer interactions. Relevant research shows that positive parenting styles promote the development of good peer relationships, while negative parenting styles negatively predict individual peer relationships (Wang et al., 2006). Positive parenting creates a relatively warm and harmonious environment for children, enhancing their communication and empathy skills, leading to more altruistic behaviors during peer interactions and better peer relationships (Li et al., 2023). Hostile and withdrawn negative co-parenting can positively predict high levels of peer conflict and low levels of positive peer interactions (Leary and Katz, 2004), which is detrimental to the formation of good peer relationships in children. Additionally, related research shows that both relational aggressors and relational aggression victims experience some psychological issues and higher levels of peer rejection. Adolescents with high levels of peer support receive more emotional support, resolve conflicts with others in reasonable ways, and exhibit less aggressive behavior (Sánchez-Moscona and Eiroa-Orosa, 2020). Based on the above, this study proposes hypothesis H2: Peer relationships mediate the relationship between parental co-parenting and relational aggression.

### The mediating role of self-control

Self-control is the ability of individuals to change their behavioral responses or internal states when faced with obstacles to their goals (Harter et al., 1982). It is a conscious and willful effort, essentially involving the individual’s exertion of “inhibition” to restrain their own behavior. This self-restraint has also been applied in the study of aggressive behavior (Xu et al., 2022). Self-control theory posits that individual behavior possesses a characteristic that can predict undesirable or deviant behaviors. The innate selfish tendencies make individuals more focused on their own interests, making it easier for those with weak moral and legal awareness to experience imbalances in self-control, leading to problematic behaviors (Guo et al., 2023). However, individuals with self-control abilities can regulate their behaviors, thoughts, and emotions, exerting willful effort to control behaviors that violate social moral norms (Baumeister et al., 2007). Previous studies have shown that individual self-control plays a moderating role between negative parenting styles and aggressive behavior (Song et al., 2017), and self-control acts as a mediating factor between parenting styles and aggressive behavior (Liu, 2022). Individuals with high self-control exhibit low aggressive behavior, while those with low self-control display high aggressive behavior (Cao and Zhang, 2018). Additionally, strengthening self-control can suppress aggressive behavior, while a decline can led to an increase in aggressive behavior (Denson et al., 2012). Enhancing self-control can improve individuals’ undesirable behaviors. Based on this, the present study proposes hypothesis H3: self-control mediates the relationship between cooperative parenting and relational aggression.

### The serial mediating role of peer relationships and self-control

**Figure 1:**
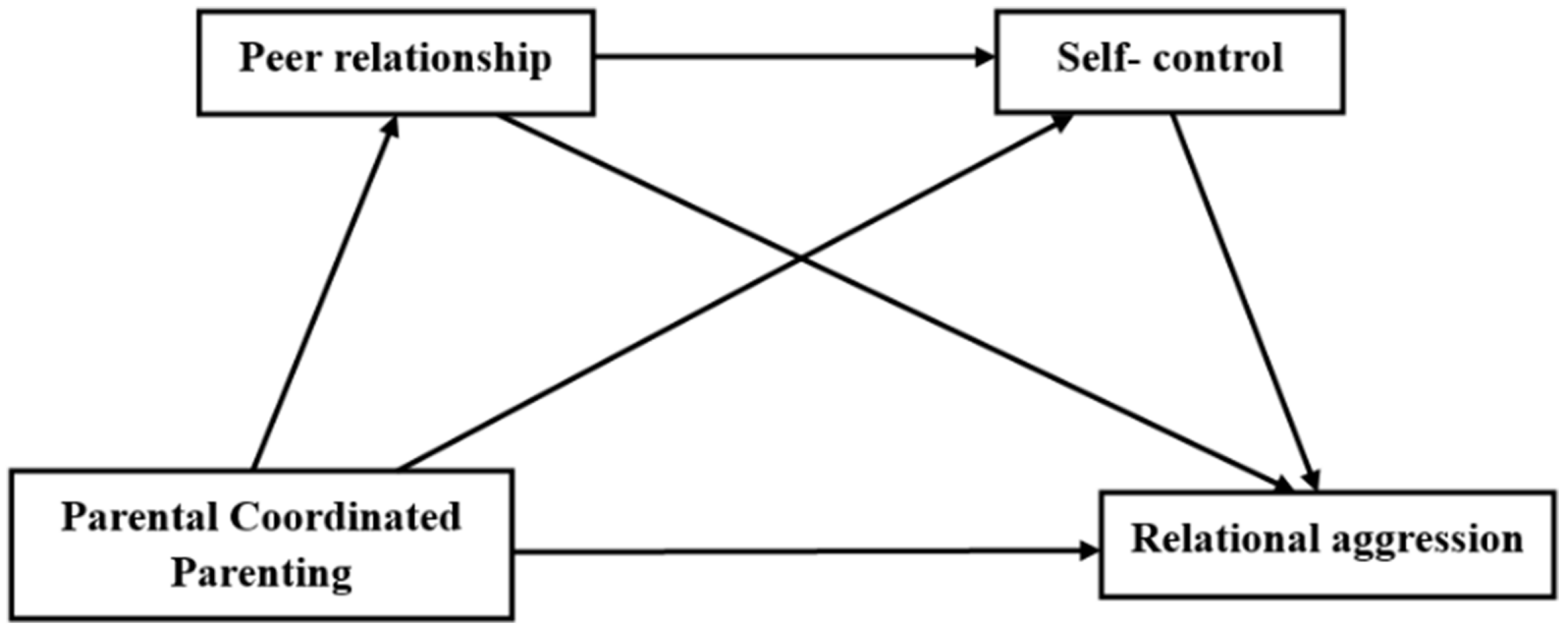
Diagram of the relationship between the variables.

Parental Coordinated Parenting may also influence adolescents’ relational aggression through the chain mediation of peer relationships and self-control. According to group social theory, peer groups are an important social environment for individuals in social interactions, where similar attitudes and behaviors can emerge through imitation (Harris, 1995). Parenting styles can be transmitted through peer interactions; positive Parental Coordinated Parenting encourages children to engage more actively in peer interactions, leading to better self-control over their behavior and a reduction in relational aggression. On one hand, positive parenting styles can have a beneficial effect on children’s peer interactions, while a positive and harmonious parenting approach creates a warm and harmonious developmental environment, fostering more altruistic behaviors during peer interactions and better peer relationships (Li et al., 2023). On the other hand, good peer interactions significantly impact students’ self-control abilities; positive peer relationships enable students to apply self-control to manage their behavior when facing problems, thereby inhibiting relational aggression (Denson et al., 2012). Therefore, students with positive collaborative parenting may have better peer relationships, which in turn enhances their self-control abilities and reduces relational aggression. Based on the above, we propose hypothesis H4: Peer relationships and self-control play a chain mediation role between Parental Coordinated Parenting and relational aggression. Figure 1 illustrates the relationship model for studying the variables.

## Methods

### Participants and procedure

The sample included 456 adolescents (51.7% male; age 13–17, *M* = 15) from three junior high schools in Tongliao, Inner Mongolia. Parental marital status was recorded: 82% of participants came from two-parent households, 12% from single-parent households, and 6% from blended families. Socioeconomic status (SES) was assessed via parental education levels (45% high school graduates, 30% college-educated). Data on divorced and single-parent families were excluded from the statistical process.

All procedures in this study were reviewed and approved by the ethics committee of the author’s university. Written informed consent was obtained from all participants and their guardians. Surveys were administered during school hours by trained researchers, with anonymity ensured to reduce social desirability bias. Data collection followed a standardized protocol, including a 10-min briefing on survey objectives and confidentiality.

### Measures

#### Parental coordinated parenting questionnaire

This study employed the Parental Coordinated Parenting Questionnaire (Adolescent Rating Version) developed by Chinese scholars Liu et al. (2017) was adopted in this study. This 29-item instrument comprises four dimensions: Unity, Consistency, Conflict, and Disparagement. (1) When I do something wrong, my dad and mom deal with it in the same way, (2) Dad (Mom) will talk about Mom’s (Dad’s) shortcomings in front of me, (3) Mom (Dad) is hostile to Dad (Mom)..., utilizing a Likert 7-point scale (1 = Never, 7 = Always). Among these dimensions, Unity and Consistency represent positive co-parenting behaviors, where higher scores indicate that the father or mother exhibited more positive parenting practices. Conversely, Conflict and Disparagement reflect negative co-parenting behaviors, with elevated scores denoting increased negative co-parenting manifestations. The Cronbach’s *α* coefficients for the paternal and maternal versions of the questionnaire in this study were 0.84 and 0.83 respectively, demonstrating satisfactory reliability.

#### Relational aggressive behavior scale

This study employed the Relational Aggression Scale originally developed by Loudin and revised by Chinese scholar Liang (2005). The 9-item instrument(1) When someone makes me angry, ignore him for a short period of time. (2) When I have a disagreement with a friend of the opposite sex, belittle him in front of a friend of the opposite sex. (3) Quarrel with someone and say that the person is not good in front of others…, utilizes a 5-point Likert scale (1 = Highly consistent, 5 = Not consistent at all) with reverse scoring implementation. Specifically, higher composite scores indicate greater relational aggression behaviors, while lower scores correspond to reduced manifestation of such behaviors. The scale demonstrated good reliability in the current investigation with a Cronbach’s *α* coefficient of 0.80.

#### Peer Relationship Scale

This study employed the Student Peer Relationship Scale originally developed by Asher (1986) and culturally adapted by Chinese scholar Zhang (2008). The 16-item instrument (1) I make new friends easily at school. (2) I have a lot of friends. (3) I am always alone…, utilizes a 4-point Likert scale (1 = Strongly disagree, 4 = Strongly agree) measuring three dimensions: Acceptance, Rejection, and Loneliness. Higher composite scores on the scale indicate better peer relationships. The measure demonstrated excellent reliability in the current study with a Cronbach’s α coefficient of 0.89.

#### Self-Control Scale

This study employed the Self-Control Scale (SCS) originally developed by Tangney et al. (2004) and culturally validated by Chinese scholar Tan and Guo (2008). The 19-item instrument (1) I can resist temptations very well. (2) It’s difficult for me to get rid of bad habits. (3) Everyone says that I have iron-like self-control…, utilizes a 5-point Likert scale (1 = Strongly disagree, 5 = Strongly agree) encompassing five dimensions: Temptation Resistance, Healthy Habits, Leisure Moderation, Impulse Control, and Work Focus. Higher composite scores across these dimensions indicate stronger self-regulation capacity. The scale demonstrated excellent psychometric properties in this investigation, with a Cronbach’s *α* coefficient of 0.88.

### Statistical processing

SPSS 24.0 software was used for data analysis, Harman’s one-way analysis of variance test for common method bias test, as well as the use of PROCESS model 6 to verify the chain mediating role of peer relationships and self-control between parental co-parenting and relational aggression. Standard errors of parameter estimates and Bootstrap confidence intervals were obtained by taking 5,000 Bootstrap samples and were considered significant if the 95% confidence interval did not include zero.

## Results

### Common method bias test

Using Harman’s one-way test, all items of the parental co-parenting, relational aggression, self-control, and peer relationship variables were analyzed in an exploratory factor analysis, and the results showed that there were 21 factors with eigenvalues greater than one, and the cumulative variation explained by the first factor was 21.5%, which was less than the critical criterion of 40%, so this study did not have a serious problem of common method bias.

### Descriptive statistics and correlation analysis

According to the correlations among the variables in Table 1, it can be seen that positive parental co-parenting and relational aggression are significantly negatively correlated; negative parental co-parenting is significantly positively correlated with relational aggression; positive parental co-parenting is significantly positively correlated with peer relationships and self-control; negative parental co-parenting is significantly negatively correlated with peer relationships and self-control; relational aggression is significantly negatively correlated with peer relationships and self-control; and peer relationships and self-control were significantly positively correlated.

**Table 1:** Descriptive statistics and correlation analysis among the variables.

Variable	M ± SD	1	2	3	4	5	6	7	8	9
1. Fathers actively coordination	67.57 ± 26.24	1								
2. Father negative coordination	26.64 ± 15.79	−0.248^**^	1							
3. Mothers are actively collaborative	68.42 ± 22.39	0.800^**^	−0.224^**^	1						
4. Mothers negative coordination	27.54 ± 15.77	−0.252^**^	0.920^**^	−0.253^**^	1					
5. Parents actively coordination	135.99 ± 46.15	0.957^**^	−0.249^**^	0.940^**^	−0.266^**^	1				
6. Parental negative coordination	54.18 ± 30.93	−0.255^**^	0.980^**^	−0.243^**^	0.980^**^	−0.263	1			
7. Relational attacks	16.22 ± 5.64	−0.118^*^	0.192^**^	−0.128^**^	0.201^**^	−0.129^**^	0.201^**^	1		
8. Peer relationships	51.09 ± 8.34	0.196^**^	−0.219^**^	0.313^**^	−0.247^**^	0.264^**^	−0.238^**^	−0.253^**^	1	
9. Self-control	63.29 ± 13.23	0.229^**^	−0.200^**^	0.331^**^	−0.241^**^	0.291^**^	−0.225^**^	−0.323^**^	0.409^**^	1

**p < 0.01, *p<0.05.

### The mediating role of peer relationships and self-control

This study employs Hayes’ non-parametric percentile Bootstrap method for testing mediation effects, specifically using the SPSS macro program PROCESS model 6. It examines the mediating effects of peer relationships and self-control on the relationship between Parental Coordinated Parenting and relational aggression, controlling for gender and age. The analysis results are shown in Table 2. the negative collaborative parenting style of parents has a significant positive direct predictive effect on relational aggression (*β* = 0.11, *p* < 0.001); positive collaborative parenting by parents significantly positively predicts self-control (*β* = 0.20, *p* < 0.001) and significantly positively predicts peer relationships (*β* = 0.26, *p* < 0.001); the negative collaborative parenting style of parents significantly negatively predicts self-control (*β* = −0.14, *p* < 0.001) and significantly negatively predicts peer relationships (*β* = −0.24, *p* < 0.001); self-control positively predicts peer relationships (*β* = 0.36, *p* < 0.001) and significantly negatively predicts relational aggression (*β* = −0.27, *p* < 0.001); peer relationships significantly negatively predict relational aggression (*β* = −0.14, *p* < 0.001).

**Table 2:** Analysis of the chain mediation model.

Variable	Self-control		Peer relationships		Relational aggression	
	*β*	*t*	*β*	*t*	*β*	*t*
Parents actively coordination	0.20	4.53^***^	0.26	5.72^***^	−0.02	−0.40
Parental negative coordination	−0.14	−3.16^***^	−0.24	−5.25^***^	0.11	2.45^**^
self-control			0.36	8.20^***^	−0.27	−5.38^***^
Peer relationships					−0.14	−2.90^***^
gender	−0.05	−0.64	−0.03	−0.38	−0.12	−1.35
age	−0.03	−1.69	−0.01	−0.27	−0.02	−0.90
*R^2^*	0.21		0.07		0.13	
*F*	29.88^***^		11.33^***^		13.19^***^	

***p < 0.001; The data of each variable have been standardized, the same below.

As shown in Table 3, the mediation effect test was conducted and found that peer relationships and self-control fully mediated between positive parental co-parenting and relational aggression, with a mediation effect value of −0.134, and the mediation effect was formed as an indirect effect generated by three paths: (1) indirect effect 1 (−0.037) generated by positive parental co-parenting → peer relationships → relational aggression; (2) indirect effect 2 (−0.053) generated by positive parental synergistic parenting → self-control → relational aggression produced indirect effect 2 (−0.053); and (3) positive parental synergistic parenting → peer relationships → self-control → relational aggression produced indirect effect 3 (−0.024). The results all indicated that the 95% confidence intervals for all three indirect paths did not include 0, suggesting that the three generated mediation effects were significant.

**Table 3:** The mediating effect of peer relationship and self-control on parental active collaborative parenting is tested.

Mediation paths	Effect size	SE	Confidence interval		
			95%CI	95%CI	Relative effects
X → M1 → Y	−0.037	0.02	−0.08	−0.01	27.61%
X → M2 → Y	−0.053	0.02	−0.09	−0.02	39.55%
X → M1 → M2 → Y	−0.024	0.01	−0.04	−0.01	17.91%
X → Y	−0.019	0.05	−0.11	0.074	14.18%
Total effect	−0.134	0.05	−0.23	−0.04	100%

X indicates parental active collaborative parenting, M1 indicates peer relationship, M2 indicates self-control, Y indicates relational aggression.

As shown in Table 4, the mediating effect test was conducted and found that the effect value of the direct effect of negative parental co-parenting on relational aggression was 0.111; peer relationships and self-control had a partially mediating role between negative parental co-parenting and relational aggression, with a mediating effect value of 0.088, which accounted for 44% of the total effect value of parental co-parenting on relational aggression (0.2). Specifically, the mediating effect was formed as an indirect effect generated by three pathways: (1) indirect effect 1 (0.03) generated by negative parental co-parenting → peer relationships → relational aggression; (2) indirect effect 2 (0.035) generated by negative parental co-parenting → self-control → relational aggression; and (3) indirect effect 3 (0.035) generated by negative parental co-parenting → peer relationships → self-control → relational aggression. Effect 3 (0.023). The results all indicated that the 95% confidence intervals of the three indirect paths did not include 0, suggesting that the three produced significant mediation effects.

**Table 4:** The mediating effect of peer relationship and self-control on parental negative collaborative parenting is tested.

Mediation paths	Effect size	SE	Confidence interval	
			95%CI	Relative effects
X → M1 → Y	0.030	0.016	[0.009, 0.065]	15%
X → M2 → Y	0.035	0.014	[0.010, 0.066]	17.5%
X → M1 → M2 → Y	0.023	0.007	[0.010, 0.039]	11.5%
X → Y	0.111	0.046	[0.022, 0.201]	55.5%
Total effect	0.20	0.046	[0.110, 0.29]	100%

X indicates parental passive collaborative parenting, M1 indicates peer relationship, M2 indicates self-control, Y indicates relational aggression.

## Discussion

The results of this study found that negative co-parenting by parents significantly positively predicts relational aggression in adolescents, confirming hypothesis H1: Parenting styles predict adolescents’ relational aggression behaviors. This is consistent with previous research findings that positive supportive co-parenting by parents can significantly reduce relational aggression behaviors in middle school students, while negative parenting styles contribute to aggressive behaviors in children and adolescents (Mukhtar and Mahmood, 2018; Xu, 2019). According to ecological systems theory and the cumulative risk model, the development of adolescent behavior is influenced by multiple systems, including social and family environments, as well as risk factors that often occur simultaneously among peers. The co-occurrence of risk factors across multiple systems constitutes cumulative ecological risk and affects adolescents’ aggressive behaviors (Hong et al., 2023). This suggests that parents should guide their children with positive parenting methods, pay attention to their emotional changes, and when children exhibit aggressive behaviors, parents should provide patient guidance rather than negative parenting, which helps children develop good peer relationships and grow up healthily both physically and mentally.

In addition, the results of this study show that positive co-parenting by fathers and positive co-parenting by mothers, as well as negative co-parenting by fathers and negative co-parenting by mothers, are significantly correlated with adolescents’ relational aggression. This is consistent with previous research findings that the co-parenting styles of parents are related to adolescents’ relational aggression. Positive co-parenting styles between parents contribute to the development of individuals at different stages, and positive co-parenting helps reduce students’ aggressive behavior (McHale, 2011). While schools and society focus on the physical and mental health development of students, they should also pay attention to the impact of family education.

Through the construction of a mediating model, it was found that peer relationships play a mediating role between parental co-parenting and relational aggression, validating research hypothesis H2. Parental co-parenting not only directly affects adolescents’ relational aggression but also influences it through the mediating role of peer relationships. This is similar to previous research findings, where negative parental co-parenting styles positively predict higher levels of peer conflict and lower levels of positive peer interactions. Adolescents with high levels of peer support tend to resolve conflicts with others in a reasonable manner and exhibit less aggressive behavior (Leary and Katz, 2004; Sánchez-Moscona and Eiroa-Orosa, 2020). The results of this study also found that parental co-parenting can influence adolescents’ relational aggression through the mediating role of self-control, validating research hypothesis H3. This is consistent with previous research findings, where children from families with negative co-parenting exhibit less behavioral inhibition, and negative parenting styles contribute to aggressive behavior in children and adolescents (Belsky et al., 1996; Mukhtar and Mahmood, 2018). Children’s self-control plays a moderating role between negative parenting styles and aggressive behavior (Song et al., 2017). Negative parenting styles affect children’s self-control abilities, leading to more aggressive behavior. Positive co-parenting styles can reduce children’s relational aggression and enhance students’ self-control abilities.

The results of the mediation effect test show that peer relationships and self-control play a chain mediation role in the impact of Parental Coordinated Parenting on adolescents’ relational aggression, validating hypothesis H4. Positive collaborative parenting by parents cannot directly predict adolescents’ relational aggression behaviors, but instead affects these behaviors through the chain mediation of peer relationships and self-control. Children experience a more positive and unified parenting style during their parents’ upbringing, which leads them to treat peer relationships positively during interactions with peers, thereby gaining more and better peers. Good peer relationships enhance students’ confidence and self-control abilities, which correspondingly reduces relational aggression behaviors. Negative collaborative parenting by parents not only directly predicts adolescents’ relational aggression behaviors but also affects relational aggression through the mediation of peer relationships and self-control. Adolescents experiencing parental conflict and negative collaborative parenting will have their peer relationships and self-control abilities affected, leading to an increase in relational aggression behaviors and more negative emotions, impacting their life and studies.

To mitigate relational aggression rooted in familial conflict, school- and community-based interventions in Inner Mongolia could integrate co-parenting workshops. Programs emphasizing conflict resolution skills and collaborative parenting strategies (e.g., the PMTO model; Forgatch and Patterson, 2010) may disrupt the intergenerational transmission of aggressive behaviors by fostering healthier marital modeling.

### The limitations and future research directions

This study explores the impact mechanism of parental co-parenting on adolescents’ relational aggression, as well as the chain mediating roles of peer relationships and self-control, enriching the relevant research. However, the data sample of this study is only from the Inner Mongolia region, so the subject group is not large enough to be generalized to other regions or countries. In addition, this study is a cross-sectional study, making it difficult to examine the longitudinal relationship between parental co-parenting and adolescents’ relational aggression, and the above results cannot help us draw causal relationships. Future studies could adopt longitudinal designs to trace dynamic changes in relational aggression across key developmental transitions, such as pre- and post-parental divorce or remarriage. Additionally, innovative methodologies like remote co-parenting assessments—for instance, analyzing interaction patterns in co-parenting apps or virtual communication logs—could provide granular insights into non-cohabiting families’ daily dynamics. Cross-cultural comparisons between Inner Mongolia and other regions with distinct ethnic compositions (e.g., Xinjiang, Tibet) may further elucidate how cultural norms modulate the co-parenting-aggression linkage.

## Conclusion

Parental co-parenting not only directly predicts adolescents’ relational aggression but can also predict adolescents’ relational aggression through the independent and chain effects of two mediating variables: peer relationships and self-control.

## Data Availability

The original contributions presented in the study are included in the article/supplementary material, further inquiries can be directed to the corresponding author.

## References

[ref0015] Asher S. R. Dodge K. A. (1986). Identifying children who are rejected by their peers. Dev. Psychol. 22, 444–449. doi: 10.1037/0012-1649.22.4.444

[ref1] Bandura A. Freeman W. H. Lightsey R. (1997). Self-efficacy: the exercise of control. J. Cogn. Psychother. 13, 158–166. doi: 10.1891/0889-8391.13.2.158, PMID: 11261958

[ref0012] Baumeister R. F. Vohs K. D. Tice D. M. (2007). The strength model of self-control. Current Directions in Psychological Science 16, 351–355. doi: 10.1111/j.1467-8721.2007.00534.x

[ref3] Belsky J. Putnam S. Crnic K. (1996). Coparenting, parenting, and early emotional development. New Dir. Child Dev. 1996, 45–55. doi: 10.1002/cd.23219967405, PMID: 9308432

[ref4] Cao X. T. Zhang L. H. (2018). The chain mediating role of adolescent emotion regulation in the relationship between self-esteem and aggression. Chin. J. Ment. Health 32, 574–579. Available at: https://kns.cnki.net/kcms2/article/abstract?v=bEegF8awJvxVL9eJUXnhpnXbrwYhOU9Q4kO6OVGy7ifvkaMf5nFyuFjil5baw6y_Ku6-JMNn5Y2nz4CSuqh6y3YNMFgtTSoxN0SL33yjI3lfFnTM0o0EJU0PZMSLZ3Z1ZMPWVBrrPjSU2OtZaPyDGym72PqYcMaFZv_6JV_gRVEm3GEMI1JSUdaZKZR22Buh&uniplatform=NZKPT&language=CHS

[ref5] Cha S. Y. (2006). Research on the influence of child temperament and relational aggression on popularity. [Master’s thesis, Zhejiang University]. CNKI. Available online at: https://kns.cnki.net/kcms2/article/abstract?v=kz9ikNiCkdrAd8GqkB49poZf5GHwP2d_4WaZ7jhQdCBf7M707L-uet5YBfe9c6Mb62a_NJly5pfpQ5vk7Fq98YdqopHtpZvLKVMD6lJAA2h4vmnOyfU2e22Z0T8pdn5gH4SSm2bJa3ZKiFhrzI_vw7CEb3IQONn6JEAeeKWqj0SQqMGO_-q-AuObSa-__55y

[ref6] Crick N. R. Grotpeter J. K. (1995). Relational aggression, gender, and social-psychological adjustment. Child Dev. 66, 710–722. doi: 10.1111/j.1467-8624.1995.tb00900.x, PMID: 7789197

[ref7] Crick N. R. Ostrov J. M. Werner N. E. (2006). A longitudinal study of relational aggression, physical aggression, and children’s social-psychological adjustment. J. Abnorm. Child Psychol. 34, 131–142. doi: 10.1007/s10802-005-9009-4, PMID: 16741683

[ref8] Denson T. F. DeWall C. N. Finkel E. J. (2012). Self-control and aggression. Curr. Dir. Psychol. Sci. 21, 20–25. doi: 10.1177/0963721411429451, PMID: 40144045

[ref0019] Forgatch M. S. Patterson G. R. (2010). “Parent Management Training—Oregon Model: An intervention for antisocial behavior in children and adolescents” in Evidence-based psychotherapies for children and adolescents 2nd ed. eds. Weisz J. R. Kazdin A. E. (The Guilford Press), 159–177. Available at: https://psycnet.apa.org/record/2010-09488-011

[ref9] Guo Q. Kang Z. Chen R. (2023). The relationship between self-control and cyber aggression in junior high school students: the mediating role of herd psychology. J. Cangzhou Norm. Univ. 40, 79–84. doi: 10.13834/j.cnki.czsfxyxb.023.01.019

[ref10] Harris J. R. (1995). Where is the child’s environment? A group socialization theory of development. Psychol. Rev. 102, 458–489. doi: 10.1037/0033-295X.102.3.458, PMID: 40095981

[ref0011] Harter S. (1982). The Perceived Competence Scale for Children. Child Development 53:87. doi: 10.2307/1129640 6525886

[ref11] Hofstede G.. (2021). Cultural hybridization in transitional societies: evidence from China's ethnic borderlands. Cross-Cult. Res. 55, 387–412. doi: 10.1177/10693971211047892

[ref12] Hong X. Liu S. Fan H. Xie H. Fang S. Zhang L. (2023). Effects of economic regional differences and family on adolescents’ aggressive behaviors: perspective of ecosystem integration. Brain Behav. 13:e2856. doi: 10.1002/brb3.2856, PMID: 36575610 PMC9927846

[ref9007] Hui L Xiayu H Xin C Lingbo Z. (2023). Parenting styles and the influence of cyberbullying on vocational students: The chain mediation effect of moraldisengagement and peer relationships. Chin. J. Clin. Psychol. 31, 114–123. doi: 10.13342/j.cnki.cjhp.2023.01.021

[ref101] Jiang L. (2016). The influence of parents ‘psychological control on relationship attacks among junior high school students (master’s thesis, Anhui Normal University). Available at: https://kns.cnki.net/kcms2/article/abstract?v=bEegF8awJvybw70N4nYf528lvvwchrvTJUGQVPmw56oAxx-yLeJi6dGNzcLXXQgm__geH7oq0XVObDEK2H_LZBUFSvflihzZTVxE1k0MEDnIgovCF-qfO5Xnmb2QzEKIDTbxQchgIabgvgKGlw40b7k942P3SWk2t-K1djjDEldKGzZUXMQ300N3jNHZtOhhy_yNz1iuTSY=&uniplatform=NZKPT&language=CHS

[ref13] Leary A. Katz L. F. (2004). Coparenting, family-level processes, and peer outcomes: the moderating role of vagal tone. Dev. Psychopathol. 16, 593–608. doi: 10.1017/S0954579404004687, PMID: 15605627

[ref9011] Lei Y Wang L Zhou Z Zhu X Dou G. (2019). The Impact of Social Exclusion on Relationship Attacks: The Role of Self-esteem and Implicit Personality Views. Chin. J. Clin. Psychol, 27, 501–505. doi: 10.16128/j.cnki.1005-3611.2019.03.0152015.01.09

[ref15] Liang F. H. (2005). Research on high school students’ relational aggression and its influencing factors [Master’s thesis, Zhejiang University]. CNKI. Available at: https://kns.cnki.net/kcms2/article/abstract?v=kz9ikNiCkdpqjWL2SC4VOSnzpTAixxOl5VLPoKDRoHxadCTHkpffJabla-Vg9vav07RAMLsv8GGl1KYW2e1qkagGr808kpx9SNGtZOXtQXxqLr_Ry0aXYIPUizBLbUiBUkNEbmn663v4SjLTdhpkryJ8XiCSfcFBKZRKnR0X5l98Wt6ovH4xAxpxOocu2_QM

[ref14] Li D. Wang H. (2021). Social normalization of indirect aggression in Chinese adolescents: a longitudinal study. Dev. Sci. 25:e13145. doi: 10.1111/desc.13145, PMID: 34224183 PMC8639626

[ref009] Li H. Huang X. Chang X. Zhao L. (2023). Parenting styles and the influence of cyberbullying on vocational students: The chain mediation effect of moral disengagement and peer relationships. J. Health Psychol. 31, 114–123. doi: 10.13342/j.cnki.cjhp.2023.01.021

[ref17] Liu C.. (2022). Interparental conflict and adolescent aggression: the mediating roles of emotional insecurity and effortful control. Dev. Psychol. 58, 1221–1236. doi: 10.1037/dev0001362 35446068

[ref16] Liu C. Wu X. C. Zou S. Q. (2017). Revision of the adolescent rating version of the parental collaborative parenting questionnaire and its reliability and validity. Chin. J. Clin. Psychol. 25, 845–849. doi: 10.16128/j.cnki.1005-3611.2017.05.012

[ref0014] Liu X. (2022). The relationship between parenting style and aggressive behavior of middle school students: the intermediary role of self-control. (master’s thesis, Hunan University of Science and Technology). doi: 10.27738/d.cnki.ghnkd.2022.000606

[ref18] Margolin G. Gordis E. B. John R. S. (2001). Coparenting: a link between marital conflict and parenting in two-parent families. J. Fam. Psychol. 15, 3–21. doi: 10.1037/0893-3200.15.1.3, PMID: 11322083

[ref19] McHale J. P. (1997). Overt and covert coparenting processes in the family. Fam. Process 36, 183–201. doi: 10.1111/j.1545-5300.1997.00183.x, PMID: 9248827

[ref20] McHale J. P. (2007). When infants grow up in multiperson relationship systems. Infant Ment. Health J. 28, 370–392. doi: 10.1002/imhj.20142, PMID: 21512615 PMC3079566

[ref21] McHale J. P. (2011). “Coparenting in diverse family systems” in Coparenting: A conceptual and clinical examination of family systems. eds. McHale J. P. Lindahl K. M. (Washington, DC: American Psychological Association), 15–37. doi: 10.1037/12328-000

[ref22] Metz M. Colonnesi C. Majdandžić M. Bögels S. M. (2017). When father steps forward and mother steps back: the moderating role of simultaneity in parents’ coparenting behaviors in the development of anxiety in 4- to 30-month-olds. Infancy 23, 103–123. doi: 10.1111/infa.12199

[ref23] Minuchin S. (1974). Families and family therapy. Harvard University Press. Available online at: https://www.hup.harvard.edu/books/9780674292369

[ref24] Mukhtar S. Mahmood Z. (2018). Moderating role of perceived social support between perceived parenting styles and relational aggression in adolescents. J. Aggress. Maltreat. Trauma 27, 831–845. doi: 10.1080/10926771.2018.1468842

[ref26] Sánchez-Moscona C. Eiroa-Orosa F. J. (2020). Training mental health peer support training facilitators: a qualitative, participatory evaluation. Int. J. Ment. Health Nurs. 30, 261–273. doi: 10.1111/inm.12781, PMID: 32893476

[ref002] Skowronski M. Weaver N. J. Wise P. S. (2005). Helping girls combat relational aggression. Communique 33, 35–37. Available at: https://www.jstor.org/

[ref27] Smith J. T.. (2022). Co-parenting in the digital age: parental phubbing and adolescent cyber-relational aggression. Comput. Hum. Behav. 136:107372. doi: 10.1016/j.chb.2022.107372, PMID: 40145060

[ref9002] Song M Chen C Liu S Li J Hou Y Zhang L. (2017). The influence of parental parenting style on adolescents’ aggressive behavior: the role of deviant peer interaction and self-control. Psychological Development and Education, 33, 675–682. doi: 10.16187/j.cnki.issn1001-4918.2017.06.05

[ref0017] Tangney J. P. Baumeister R. F. Boone A. L. (2004). High self-control predicts good adjustment, less pathology, better grades and interpersonal success. J. Pers. 72, 271–324. doi: 10.1111/j.0022-3506.2004.00263.x 15016066

[ref28] Tan S. H. Guo Y. Y. (2008). Revision of college students’ self-control scale. Chin. J. Clin. Psychol. 16, 468–470. Available at: https://kns.cnki.net/kcms2/article/abstract?v=bEegF8awJvxxU6T0-j5i9lEjDsUnmleoTnP5jny4h9dHX07BNfQwcb1IaKBqQw88qBM9-p4pE4RFc1MquKJCd-87va_qAP_bWslIbdrLfszhwrYVi06mh5yYPALQu3ZFUMbGt_1uzLQ__nOZNIcS8S9kCqdBFAdIoRI-7K6zlknUnKTE-B_tGkA3urW8bWDn&uniplatform=NZKPT&language=CHS

[ref29] Wang W. C. Tan X. Q. (2016). Research status of children’s relational aggression in China from 2004 to 2014. Chin. J. Sch. Health 37, 538–542. doi: 10.16835/j.cnki.1000-9817.2016.04.019

[ref9003] Wang Z Luo H Zhang J. (2006). The relationship between parental parenting style and personality characteristics of adolescents. Chin. J. Clin. Psychol. 315–317. Available at: https://kns.cnki.net/kcms2/article/abstract?v=bEegF8awJvzbwRbgx9cFfKzwx6BLro3ALXUXwLEGDhuYsEa3ODvapMglMTFYsDTb6pItYLEZj4ad7r3cXIygm8OFv3yZ7bIgdt29TVUuOzsZXN4q_jaimi8O3KxeY5PAoF2ER8jfAn87jzguwcBwTMvgZrlOXUFW_63ipvUnrX81yT-uPfvkP_wyYXvxS29I\u0026amp;uniplatform=NZKPT\u0026amp;language=CHS

[ref30] Xu J. Y. Chu K. Q. Zhu H. Zhu F. S. (2022). The relationship between college students’ self-efficacy and aggression behavior: the mediating role of self-control and the moderating role of gender. Campus Psychol. 20, 415–420. doi: 10.19521/j.cnki.1673-1662.2022.06.003

[ref0001] Xu Y. (2019). Collaborative parenting and high school students aggressive behavior- -the mediation role of cognitive emotion regulation strategy (master’s thesis, Liaoning Normal University). Available at: https://kns.cnki.net/kcms2/article/abstract?v=bEegF8awJvx7cUosJqeRqGDZzyQutszGDPNwz2OWzfsouTxRfe4sUEhbrmC1F4Q4FLCeJxl5irpDN2HM4PVNG3HwGtGNA1aTijEd7xcxv-4CWgZkdl4ws1Ob5eB46YL9O_3AAsmXQrCu8lGoFT9w6fJcYUAT1AqO0SKfe40XVTttBvt50M6tuFrtSyK2tKWEy4nGOvqKq2Y=&uniplatform=NZKPT&language=CHS

[ref9015] Yujie L. Lin W. Zongkui Z. Xiaowei Z. Gang D., (2019). The Impact of Social Exclusion on Relationship Attacks: The Role of Self-esteem and Implicit Personality Views. Chin. J. Clin. Psychol, 27, 501–505. doi: 10.16128/j.cnki.1005-3611.2019.03.015

[ref001] Zhang Y. (2008). A Study on the Relationship between Junior High School Students ‘Self-concept and School Adaptation (Master’s thesis, Northwest University). Available at: https://kns.cnki.net/kcms2/article/abstract?v=bEegF8awJvy4ALMTQhrwrM70MTeZAYCNG1yXCGtZCg4L6ujP6dlaUzIn5uz79IwTbe_M_E960im5Xz3l1s1JC3BiquYDr7fHBM_P8YgeQMoHq6JzY0_jRumvhgDHvchlemZqR2qgN5jn_t-QC9NK9nKbLvElmeKyffc2LMkFknTfvGA10aGB2aJ7sx_EJKwT&uniplatform=NZKPT&language=CHS

[ref9005] Zhou Z Sun X Zhao D Tian Y Fan C. (2015). A Study on the Development of Peer Relationships. Psychological Development and Education, 31, 62–70. doi: 10.16187/j.cnki.issn1001-4918

[ref007] Zongkui Z. Xiaojun S. Dongmei Z. Yuan T. Cuiying F. (2015). A Study on the Development of Peer Relationships. Psychological Development and Education 31, 62–70. doi: 10.16187/j.cnki.issn1001-4918.2015.01.09

